# Neurotology: definitions and evidence-based therapies – Results of the I Brazilian Forum of Neurotology^☆^

**DOI:** 10.1016/j.bjorl.2019.11.002

**Published:** 2019-12-03

**Authors:** Márcio Cavalcante Salmito, Francisco Carlos Zuma e Maia, Mário Edvin Gretes, Alessandra Venosa, Fernando Freitas Ganança, Maurício Malavasi Ganança, Raquel Mezzalira, Roseli Saraiva Moreira Bittar, Alexandre Camilotti Gasperin, Anna Paula Batista de Ávila Pires, Bernardo Faria Ramos, César Bertoldo, Cícero Ferreira, Danilo Real, Humberto Afonso Guimarães, Jeanne Oiticica, Joel Lavinsky, Karen Carvalho Lopes, Juliana Antoniolli Duarte, Lígia Oliveira Gonçalves Morganti, Lisandra Megumi Arima dos Santos, Lúcia Joffily, Luíz Lavinsky, Mônica Alcantara de Oliveira Santos, Patrícia Mauro Mano, Pedro Ivo Machado Pires de Araújo, Pedro Luís Mangabeira Albernaz, Renato Cal, Ricardo Schaffeln Dorigueto, Rita de Cássia Cassou Guimarães, Rogério Castro Borges de Carvalho

**Affiliations:** a Universidade Federal de São Paulo (Unifesp), Escola Paulista de Medicina, Departamento de Otorrinolaringologia e Cirurgia Cérvico-Facial, Disciplina de Otologia e Otoneurologia, São Paulo, SP, Brazil; b Pontifícia Universidade Católica do Rio Grande do Sul (PUC-RS), Porto Alegre, RS, Brazil; c Pontifícia Universidade Católica de Campinas (PUC-Campinas), Faculdade de Medicina, SP, Brazil; d Universidade de Brasília (UnB), Brasília, DF, Brazil; e Universidade de Campinas (Unicamp), Disciplina de Otorrinolaringologia Cabeça e Pescoço, Campinas, SP, Brazil; f Hospital das Clínicas da Faculdade de Medicina da Universidade de São Paulo (HCFMUSP), Setor de Otoneurologia, São Paulo, SP, Brazil; g Instituto Paranaense de Otorrinolaringologia (IPO-PR), Serviço de Otologia Clínica, Curitiba, PR, Brazil; h Universidade Federal de Minas Gerais (UFMG), Belo Horizonte, MG, Brazil; i Universidade Federal do Espírito Santo (UFES), Vitória, ES, Brazil; j Hospital Mater Dei, Belo Horizonte, MG, Brazil; k Universidade Federal do Rio Grande do Sul (UFRGS), Porto Alegre, RS, Brazil; l Universidade Federal de Ciências da Saúde de Porto Alegre (UFCSPA), Santa Casa de Porto Alegre, Porto Alegre, RS, Brazil; m Universidade Federal de São Paulo (Unifesp), São Paulo, SP, Brazil; n Universidade Federal de Minas Gerais (UFMG), Faculdade de Medicina, Departamento de Otorrinolaringologia, Belo Horizonte, MG, Brazil; o Hospital Universitário Gaffrée e Guinle, Universidade Federal do Estado do Rio de Janeiro (Unirio), Rio de Janeiro, RJ, Brazil; p Universidade Federal do Rio Grande do Sul (UFRGS), Faculdade de Medicina, Disciplina de Otorrinolaringologia, Porto Alegre, RS, Brazil; q Irmandade da Santa Casa de Misericórdia de São Paulo, Faculdade de Ciências Médicas, Departamento de Otorrinolaringologia, São Paulo, SP, Brazil; r Instituto de Assistência Médica ao Servidor Público Estadual de São Paulo, São Paulo, SP, Brazil; s Hospital Federal dos Servidores do Rio de Janeiro, Departamento de Otorrinolaringologia e Cirurgia de Cabeça e Pescoço, Rio de Janeiro, RJ, Brazil; t Universidade Federal de Goiás (UFG), Goiânia, GO, Brazil; u Hospital Israelita Albert Einstein, São Paulo, SP, Brazil; v Universidade Federal do Pará (UFPa), Faculdade de Medicina, Belém, PA, Brazil; w Hospital Paulista, São Paulo, SP, Brazil; x Universidade Federal do Paraná (UFPR), Curitiba, PR, Brazil; y Clínica Borges de Carvalho Otorrinos, Rio de Janeiro, RJ, Brazil

**Keywords:** Vertigo, Dizziness, Neurotology, Vertigem, Tontura, Otoneurologia

## Abstract

**Introduction:**

Neurotology is a rapidly expanding field of knowledge. The study of the vestibular system has advanced so much that even basic definitions, such as the meaning of vestibular symptoms, have only recently been standardized.

**Objective:**

To present a review of the main subjects of neurotology, including concepts, diagnosis and treatment of Neurotology, defining current scientific evidence to facilitate decision-making and to point out the most evidence-lacking areas to stimulate further new research.

**Methods:**

This text is the result of the I Brazilian Forum of Neurotology, which brought together the foremost Brazilian researchers in this area for a literature review. In all, there will be three review papers to be published. This first review will address definitions and therapies, the second one will address diagnostic tools, and the third will define the main diseases diagnoses. Each author performed a bibliographic search in the LILACS, SciELO, PubMed and MEDLINE databases on a given subject. The text was then submitted to the other Forum participants for a period of 30 days for analysis. A special chapter, on the definition of vestibular symptoms, was translated by an official translation service, and equally submitted to the other stages of the process. There was then a in-person meeting in which all the texts were orally presented, and there was a discussion among the participants to define a consensual text for each chapter. The consensual texts were then submitted to a final review by four professors of neurotology disciplines from three Brazilian universities and finally concluded. Based on the full text, available on the website of the Brazilian Association of Otorhinolaryngology and Cervical-Facial Surgery, this summary version was written as a review article.

**Result:**

The text presents the official translation into Portuguese of the definition of vestibular symptoms proposed by the Bárány Society and brings together the main scientific evidence for each of the main existing therapies for neurotological diseases.

**Conclusion:**

This text rationally grouped the main topics of knowledge regarding the definitions and therapies of Neurotology, allowing the reader a broad view of the approach of neurotological patients based on scientific evidence and national experience, which should assist them in clinical decision-making, and show the most evidence-lacking topics to stimulate further study.

## Resumo

**Introdução:**

A otoneurologia é uma área de conhecimento que tem se expandido muito rapidamente. O estudo do sistema vestibular tem avançado tanto que mesmo definições básicas, como o significado dos sintomas vestibulares, foram apenas recentemente padronizadas.

**Objetivo:**

Apresentar uma revisão dos principais assuntos da otoneurologia, inclusive conceitos, diagnóstico e tratamento da otoneurologia, definir a evidência científica atual para facilitar a tomada de decisões e demonstrar as áreas mais carentes de evidência para estimular novas pesquisas.

**Método:**

Este texto é fruto do I Fórum Brasileiro de Otoneurologia, que reuniu os principais pesquisadores brasileiros dessa área para uma revisão da literatura. Serão feitos três trabalhos de revisão a serem publicados. Este primeiro abordou as definições e as terapias, o segundo abordará as ferramentas diagnósticas e o terceiro definirá os principais diagnósticos. Cada autor fez um levantamento bibliográfico na base de dados da Lilacs, SciELO, Pubmed e Medline de um determinado assunto. O seu texto foi então submetido aos demais participantes do Fórum por 30 dias para análise. Um capítulo especial, da definição dos sintomas vestibulares, foi traduzido por serviço de tradução oficial e igualmente submetido às demais etapas do processo. Houve então uma reunião presencial em que todos os textos foram apresentados oralmente e houve uma discussão entre os participantes para a definição de um texto consensual para cada capítulo. Os textos consensuais foram então submetidos a uma revisão final por quatro professores de otoneurologia de três universidades brasileiras e, por fim, finalizado. A partir do texto completo, publicado no site da Associação Brasileira de Otorrinolaringologia e Cirurgia Cérvico-Facial, foi escrita esta versão-resumo como artigo de revisão.

**Resultado:**

O texto apresenta a tradução oficial para o português da definição dos sintomas vestibulares propostos pela *Barany Society* e agrupa as principais evidências científicas para cada um das principais terapias existentes para as doenças otoneurológicas.

**Conclusão:**

Este texto agrupou de forma racional os principais tópicos de conhecimento a respeito das definições e terapias da otoneurologia, permite ao leitor uma visão ampla da abordagem dos pacientes otoneurológicos baseada em evidências científicas e experiência nacional, que deverá auxiliá-lo na tomada de decisões clínicas, e mostra os assuntos mais carentes de evidência para estimular novos estudos.

## Introduction

Neurotology is a medical discipline that explores the interface between otorhinolaryngology and neurology, comprising the clinical evaluation and treatment of sensorineural hearing and balance disorders. In recent years, new knowledge and new test modalities have been incorporated into neurotology. In order to gather the scientific evidence of neurotology into an easily accessible and succinct document, the ABORL-CCF, through its Department of Neurotology, developed the Neurotology Forums project to present to doctors who treat neurotological diseases an organized meeting of scientific evidence, facilitating the management of the neurotological patient.

## Methods

This text was the result of the I Neurotology Forum, held with the leading specialists in the field in Brazil on September 2, 2017. Prior to the meeting, the texts had been written based on a literature review to gather scientific evidence divided by topics. For this first Forum, the topic definitions and therapies was chosen. In addition to a literature review, contact was made with the Barany Society, which authorized the official translation of the concepts defined in the world consensus of the international classification of vestibular diseases,^1^ which is published in its entirety in the Forum text, on the website of the Brazilian Association of Otorhinolaryngology and Cervicofacial Surgery (ABORL-CCF).

The texts written by the experts were then screened by the other Forum participants a few weeks in advance. Each author presented their text on the day of the meeting and, after debating them among those present, the texts were finalized by consensus. The texts were then grouped and standardized. On October 27, 2018, at a final meeting with representatives of the Neurotology training services in the country, the text was read again, and minor final adjustments were made. The recommendations of this first Forum, based on the literature, are objectively described and summarized in this article. For further reading, the reader may refer to the Forum text published in full on the ABORL-CCF website.

## Results

### Medical competencies in neurotology

The physician must respect the legal determinations and guidelines dictated by the Federal Council of Medicine (CFM) and ABORL-CCF. The teaching of private medical acts in any form of knowledge transmission to non-medical professionals, including those pertaining to advanced life support, except remote emergency care, is prohibited until optimal resources are reached (*Resolution of the Federal Council of Medicine 1718/2004, ratified by ABORL-CCF at the ABORL-CCF Ordinary General Meeting*).

The medical report with diagnosis is the exclusive competency and prerogative of the physician, who is responsible for diagnosing the diseases and prescribing treatments, whereas the other professionals will act only within the scope of their respective legislations, according to the jurisprudence of the Superior Courts. People who perform acts of diagnosis of diseases and prescription of treatments should be reported to the authorities for illegal practice of Medicine, a crime provided for in the Penal Code with penalties ranging from six months to two years in prison (*Note from the Federal Council of Medicine on 08/21/2013*).

The professionals that comprise the multidisciplinary team can perform the procedures prescribed by the medical professional within the limits of their competency. Medical consultations, which include anamnesis, physical examination, and creation of diagnostic hypotheses and indication of treatment that encompass the medical act are the exclusive attributions of the medical professional and, therefore, to establish the diagnosis of the diseases is an exclusive medical prerogative.

Considering the increasing complexity of medical problems, it is essential the team of physicians and other health professionals, such as nurses, pharmacists, physiotherapists and speech therapists, respect the limits of their competencies, prerogatives and their strict professional scope (*Technical Note of the Department of Neurotology at ABORL-CCF 08/01/2017*).

### Definitions of vestibular symptoms^1^

#### Vertigo (in Portuguese: vertigem)

(Internal) vertigo is the sensation of self-motion when no self-motion is occurring or the sensation of distorted self-motion during an otherwise normal head movement. The term encompasses false spinning sensations (spinning verti- go) and also other false sensations like swaying, tilting, bobbing, bouncing, or sliding (non-spinning vertigo).

#### Dizziness (in Portuguese: tontura)

(Non-vertiginous) dizziness is the sensation of disturbed or impaired spatial orientation without a false or distorted sense of motion.

#### Vestibulo-visual symptoms (in Portuguese: sintomas vestíbulo-visuais)

Vestibulo-visual symptoms are visual symptoms that usually result from vestibular pathology or the interplay between visual and vestibular systems. These include false sensations of motion or tilting of the visual surround and visual distortion (blur) linked to vestibular (rather than optical) failure. There are five vestibulo-visual symptoms: *external vertigo, oscillopsia, visual lag, visual tilt, and movement-induced blur.*

#### Postural symptoms (in Portuguese: sintomas posturais)

Postural symptoms are balance symptoms related to maintenance ofpostural stability, occur- ring only while upright (seated, standing, or walking). There are four postural symptoms: *Unsteadiness, directional pulsion, balance-related near-fall, and balance-related fall*.

### Definition of vestibular syndromes

Clinical history is the main tool in the search for patient diagnosis. According to the Bárány Society, vestibular syndromes have been classified into 3 distinct groups: acute, episodic, and chronic.^2^

#### Acute vestibular syndromes (AVS)

These are characterized by the abrupt onset of vestibular symptoms that persist for days or weeks, commonly associated with nausea, vomiting, gait unsteadiness, movement intolerance and presence of nystagmus. After the initial peak of symptoms, there occurs improvement during the first week and gradual recovery over weeks to months. AVS may occur spontaneously, following trauma or exposure to toxic agents, among others.^3^ The main diagnoses are acute unilateral vestibular hypofunction (vestibular neuritis), hemorrhagic or ischemic cerebrovascular accident (CVA) in the posterior fossa, labyrinthitis, head trauma and vestibulotoxicity.

#### Episodic vestibular syndromes

These present as recurrent episodes of vestibular symptoms lasting from seconds to hours. The inter-crisis period may be asymptomatic or maintain some degree of milder dizziness in relation to the crises.^4^ They can be subdivided into spontaneous (Meniere’s disease, vestibular migraine, transient ischemic attack (TIA) of the posterior circulation) and triggered (benign paroxysmal positional vertigo (BPPV), perilymphatic fistula, superior semicircular canal dehiscence (SSCD) syndrome, vertebrobasilar insufficiency syndrome, medication side effects, and central positional vertigo (CPV).^2^

#### Chronic vestibular syndromes (CVS)

They are characterized by the persistence of vestibular symptoms over a long period of time. The main diagnoses are persistent postural-perceptual dizziness (PPPD), chronic idiopathic unilateral vestibulopathy, persistent unilateral vestibulopathy after vestibular neuritis, unilateral vestibulopathy secondary to vestibular schwannoma, unilateral vestibulopathy after medical procedure, chronic bilateral vestibulopathy. Other diagnoses: CANVAS, posterior fossa tumors.^5^ The Bárány Society does not specify the minimum duration from symptom onset to define CVS.

### Medications in neurotology

#### Vestibular suppressors

They may be prescribed in the acute phase of vestibular diseases for symptomatic relief. Their use should be as short as possible so as not to impair the vestibular compensation and avoid unwanted side effects. The main classes of medications and their mechanisms of action are:

• **Calcium channel antagonists**: sedative action, as they antagonize slow calcium channels and also because they have antihistamine action on H1 receptors. In addition to these effects, peripheral vasodilation action is described.^6^ The most commonly used medications are flunarizine and cinnarizine.

Level of Evidence: *B*.

Degree of recommendation: *recommended*.

• **Antihistamines**: vestibular sedatives with anti-cholinergic and antihistamine effect on H1 receptor. They can be used in acute vertigo, for the relief of nausea and vomiting,^7^ after repositioning maneuvers and in the treatment of kinetosis. The most commonly used medications are dimenhydrinate,^8^ meclizine^9^ and promethazine.^10^

Level of Evidence: B.

Degree of recommendation: *recommended*.

• **Benzodiazepines**: vestibular sedatives because they potentiate the inhibitory action of GABA. They may also be used in acute vertigo^11^ and kinetosis prophylaxis.^12^ The most commonly used drugs are clonazepam and diazepam.

Level of Evidence: *B*.

Degree of recommendation: *recommended*.

#### Non-supressor vestibular medications

They are indicated for symptom control and prophylaxis of new crises of vertigo. The main medications in this class and their mechanisms of action are:

• **Betahistine**: it is a histaminergic modulator acting as a weak H1 agonist and a strong H3 heteroreceptor antagonist. Its action in the labyrinth involves mechanisms that facilitate fluid circulation in the stria vascularis via the precapillary sphincter, thereby reducing endolymphatic pressure.^13^ Its action on the CNS occurs by facilitating vestibular compensation and reducing bioelectric activity in the vestibular nuclei, with the latter being dose dependent.^13^, ^14^

Level of Evidence: *A*.

Degree of recommendation: *recommended*.

• **Ginkgo biloba EGb761 Extract**: it is an herbal medicine, consisting of two active fractions: flavonoids and terpenoids. It has antiplatelet, antioxidant, antihypoxemic, anti-free radical, and antiedema actions in both the central nervous system and the inner ear.^15^, ^16^

Level of Evidence: *B*.

Degree of recommendation: *recommended*.

##### Antidepressants

They are used for the prophylaxis of vestibular migraine crises and Meniere’s disease and treatment of PPPD. The start of the therapy should be gradual to assess possible adverse reactions and treatment should be continued for at least 6 months after symptom control. Withdrawal, when indicated, should also be gradual to avoid withdrawal reactions by the patient.

There are at least seven types of neurotransmitters involved in the transmission of vestibular system impulses. The top seven are glutamate, acetylcholine, GABA, dopamine, norepinephrine, histamine and serotonin. Most antidepressant medications work by modulating the action of serotonin. Serotonin receptors are found in the peripheral vestibular system and the vestibular nucleus in the central nervous system.^17^ The antidepressants that inhibit the selective serotonin reuptake may reduce tinnitus symptoms through direct inhibition of electrical impulse transmission.^18^

Listed below are the recommendations of each antidepressant according to the diagnosis:

• **Prophylaxis of vestibular migraine crises**: Nortriptyline (*C, recommended*), Amitriptyline (*C, recommended*), Venlafaxine (*B, recommended*).
• **Prophylaxis of Meniere's syndrome crises**: Sertraline (*D, optional*), Escitalopram (*C, recommended*).
• **Treatment of persistent postural-perceptual dizziness**: Sertraline (*C, recommended*), Paroxetine (*C, recommended*), Imipramine (*C, recommended*), Fluvoxamine (*C, recommended*), Milnacipran (*C, recommended*), Fluoxetine (*D, optional*), Citalopram (*D, optional*).

#### Anticonvulsants

They are used in the treatment of vestibular paroxysms and prophylaxis of vestibular migraine crises and in the control of tinnitus. The start of the therapy should be gradual to assess possible adverse reactions. Withdrawal, when indicated, should also be gradual to avoid withdrawal reactions by the patient.

Anticonvulsants are classified according to their site of action and potency: enhancers of GABA-mediated synaptic inhibition (inhibit GABA-degrading transaminase, or are direct GABAergic agonists), sodium channel blockers (reduce electrical excitability of cell membranes), calcium channel blockers (act in maintaining electrical firing), inhibitors of glutamate synaptic receptors (lower the excitability threshold). The most commonly used are carbamazepine, oxcarbazepine, topiramate, valproate, gabapentin and lamotrigine.

Anticonvulsants with their degree of recommendation, according to diagnosis, are listed below:^19^, ^20^

• **Treatment of vestibular paroxysm**: Carbamazepine (*C, recommended*), Oxcarbazepine (*C, recommended*), Gabapentin (*D, optional*), Phenytoin (*D, optional*), Valproic Acid (*C, recommended*).
• **Prophylaxis of vestibular migraine crises**: Topiramate (*C, recommended*), Valproate (*D, optional*), carbamazepine (*D, optional*), Gabapentin (*D, optional*), Lamotrigine (*D, optional*).

#### Other medications acting in vestibular diseases

They help control vestibular symptoms, fluctuating hearing, aural fullness and tinnitus in patients with Meniere’s disease.

• **Diuretics:** they can be used to treat Meniere’s disease, with cases being assessed individually. Although several experimental and radiological studies demonstrate the reversal of hydrops with the use of diuretics, the mechanism of action of these medications is still much discussed.^21^ The most studied medications are chlorthalidone, which is a thiazide diuretic, acting on the distal tubule^22^, ^23^ and acetazolamide, which is a carbonic anhydrase inhibitor.^24^

Level of Evidence: *C*.

Degree of recommendation: *optional*.

### Procedures in neurotology

#### Otolith repositioning maneuvers

These are distinct therapeutic procedures for vestibular rehabilitation exercises. While rehabilitation exercises, such as Cawthorne-Cooksey or Brandt-Daroff protocols, aim at mechanisms of neuronal plasticity such as adaptation, habituation and sensory substitution, otoliths repositioning maneuvers aim to effectively release and reposition otoliths out of the endolymphatic duct and ampoule and back to their physiological utricular position. They should be carried out by the doctor to resolve the positional vestibular symptoms in BPPV.

Level of Evidence: *A*.

Degree of recommendation: *strong*.

**Fig. 1:**
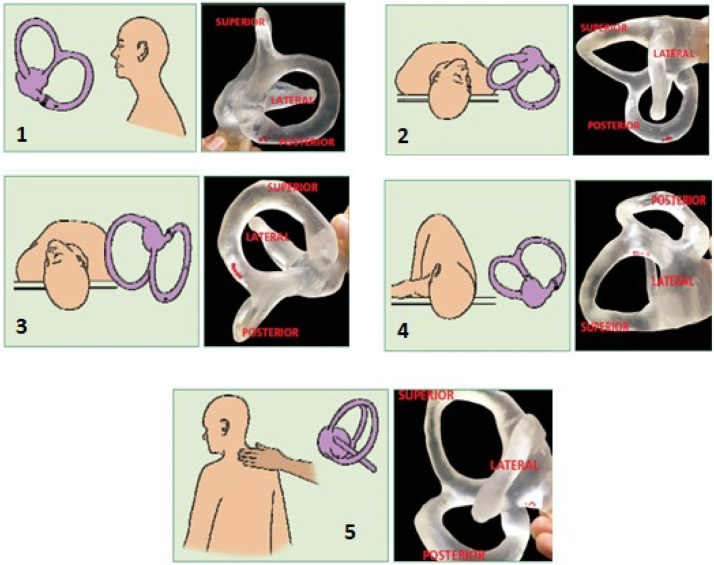
Epley Maneuver Sequence beginning at the right ear.^26^

**Fig. 2:**
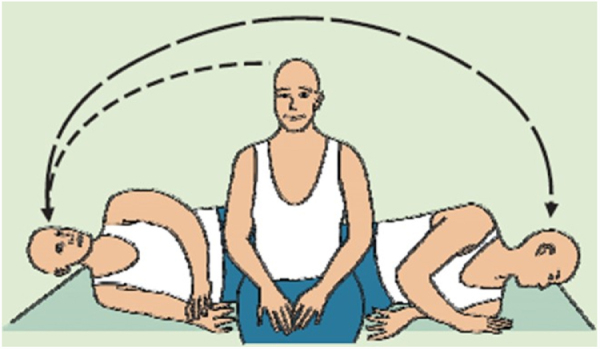
Semont releasing maneuver towards the left for the treatment of right posterior canal cupulolithiasis.^26^
• **Maneuvers for the lateral canal - canalithiasis (geotropic variant)**:^27^ there are 3 main particle repositioning methods for treating the lateral canal BPPV in the geotropic variant: the Lempert maneuver, forced and prolonged positioning, and the Gufoni maneuver. The Lempert maneuver is the most used at the moment of the bibliographic survey for this work. The patient is made to lie in the supine position, then rotates with the head turned 45 degrees towards the affected side. The patient is then taken through a series of 90-degree steps towards the unaffected side remaining in each position for 10–30 seconds, completing a 360° turn and returns to the supine position in preparation for a rapid and simultaneous face-up movement to the sitting position (Fig. 3).

• **Maneuvers for the posterior canal**:^25^ there are two main therapeutic maneuvers for posterior canal BPPV: the Epley maneuver and the Semont maneuver. In Epley maneuver, the patient is placed in the Dix Hallpike diagnostic position and remains so until nystagmus and dizziness subside, for 1−2 min. The head is slowly rotated 90° to the opposite side, being held in this position for 1−2 min more. The body is rotated to the lateral decubitus position, followed by a 90-degree movement of the head until the nose points to the ground at a 45-degree angle from the ground plane. This position is held for 30–60 seconds, and then the patient is asked to place their chin on their chest and to sit slowly. The head remains low for a moment before returning to the normal position. The sequence of movements can be seen in Fig. 1. The Semont maneuver is indicated for the treatment of cupulolithiasis of the vertical canal. When the posterior canals are involved, the patient is initially seated with the legs hanging and then moved to the lateral decubitus position of the affected side, with the head forming a 45° angle with the stretcher. Nystagmus and/or vertigo occur, and the position is maintained for 1−3 min. The examiner holds the patient’s head and neck, moving it quickly toward the other side of the stretcher. From the beginning to the end of the trajectory, the head is kept in the same position until it reaches the opposite side, when the patient touches the stretcher with their forehead and the nose (Fig. 2). In the case of the superior canals, the movement is performed in the opposite direction to that used for the maneuver of the posterior canals.

**Fig. 3:**
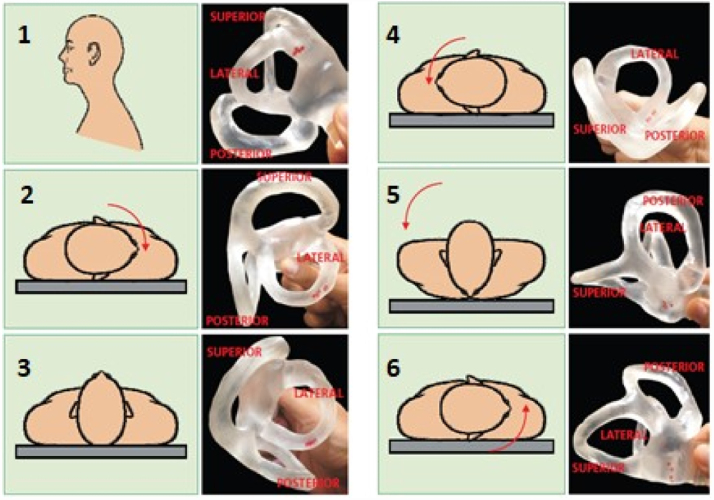
Lempert maneuver for right lateral canal treatment.^26^
• **Maneuvers for the lateral canal - cupulolithiasis (apogeotropic variant)**:^28^, ^29^ there is the Gufoni maneuver and, published more recently, the Zuma maneuver, used for both the repositioning of the particles located in the dome next to utricle on the canal side or in the anterior arm of the semicircular canal.
• **Maneuvers for the anterior canal**: these were not discussed in the forum since anterior canal BPPV is a very rare and uncertain diagnosis and its pathophysiology is not well understood.^30^
• **Complications of repositioning maneuvers**: severe complications are rare but should not be underestimated. Observation of the patient’s risk factors, and the use of appropriate techniques help to prevent them, and their performance under medical supervision is necessary for the treatment. The main complications are *severe vertigo crisis*, with nausea, vomiting and sweating, with frequent need for medication, *conversion to another semicircular canal* (the most common being the conversion of the posterior semicircular canal to the lateral canal), requiring an adequate diagnosis of the new affected canal with indication of another therapeutic maneuver, *intracanalicular otolith obstruction* (*canalith jam*) requiring reversal of position, osteoarticular and vascular cervical lesions, and endolymphatic hydrops.^31^, ^32^

#### Intratympanic injections

Used to control vestibular symptoms in Meniere’s disease and to recover rapidly progressive sensorineural hearing loss. Gentamicin application is reserved for the treatment of Meniere’s disease unresponsive to clinical treatment. Because of the possibility of hearing loss, cochlear function should be monitored. Intratympanic corticosteroid therapy can be used for sudden sensorineural hearing loss, immune-mediated inner ear diseases, and rapidly progressive sensorineural hearing loss, as primary, combined, or rescue therapy to oral corticosteroid therapy.^33^, ^34^

Level of evidence: B (vertigo control) and C (hearing loss).

Degree of recommendation: *recommended*.

#### Vestibular rehabilitation

It is a physiological form of treatment of peripheral and/or central vestibular symptoms, allowing patients to perform their usual activities to the best of their capacities. It is based on eye, head and body exercise protocols. The exercises use vestibular adaptation mechanisms, substitution of sensory or motor strategies and habituation to accelerate vestibular compensation and regain body balance.^35^, ^36^

• **Traditional**: vestibular adaptation exercises intensify vestibular-ocular reflex gain and tolerance to head movements, improve gaze stability, vestibulo-visual interaction during head movements, and postural stability in conflicting sensory environments. Sensory substitution exercises seek to intensify residual vestibular function and replace the reduced or absent vestibular function with alternative strategies of gaze stabilization and static and dynamic postural control. On the other hand, habituation exercises aim to desensitize the movements and/or positions that trigger vestibular symptoms through repetitive stimuli.

Level of Evidence: *A*.

Degree of recommendation: *strong*.

• **With platforms**: the use of platforms may be a therapeutic option included in the vestibular rehabilitation protocol. Platforms are equipment where the individual remains in an upright position, being submitted to body stability challenges caused by movements in the mid-lateral or anteroposterior direction or with flexion and extension of the ankle. There are studies showing more significant improvement in the Dizziness Handicap Inventory (DHI) and body sway scores in patients treated with the platform, compared to conventional vestibular rehabilitation.^37^ However, other studies found no significant difference between the two treatments.^38^

Level of Evidence: *C*.

Degree of recommendation: *recommended*.

• **Vibrotactile substitution**: the use of vibrotactile substitution equipment may be a therapeutic option included in the vestibular rehabilitation protocol. It is a technique that accelerates central compensation through vestibular rehabilitation by substitution. It consists of placing a vibrating device on the patient, most commonly an adjustable waist belt, which consists of a main unit, responsible for detecting movement sways, and four vibratory units, located in the anterior, posterior, right lateral and left lateral positions,^39^ which vibrate when there is inadequate body deviation and provide better postural correction.^40^, ^41^

Level of Evidence: *B*.

Degree of recommendation: *recommended*.

• **Virtual Reality**: aims to recreate environmental changes by stimulating sensory systems to adjust the reflexes involved in postural control and balance strategies.^42^ It utilizes virtual reality devices that enable immersion in an illusory world, where the perception of the environment is modified by an artificial sensory stimulus, which can cause a vestibulo-visual conflict and change in VOR gain.^42^ Compared to the conventional vestibular rehabilitation (Cawthorne and Cooksey exercises), virtual reality rehabilitation showed earlier improvement results, considering scores such as DHI, visual analog scale, and computerized posturography, in addition to the lower frequency of sessions.^43^

Level of Evidence: *B*.

Degree of recommendation: *recommended*.

• **Neuromodulation**: vestibular rehabilitation through neuromodulation may be performed in patients with peripheral or central chronic vestibular dysfunction for at least 1 year who have performed and completed previous vestibular rehabilitation therapy protocol with limited results, and whose symptoms and limitations to perform activities of daily living still persist.^44^ Neuromodulation promotes neural modulation by direct electrical or chemical stimulation in neural circuits in the brain, spinal cord, and peripheral nerves, restoring neural balance. It uses a portable device that induces neuroplasticity through noninvasive electrical stimulation of 4 cranial nerves: trigeminal, facial, glossopharyngeal and hypoglossal. It is capable of neuromodulating subcortical areas of restricted accessibility, including brainstem and cerebellum. The device should be placed and supported on the tongue; it generates electrical stimulation on the dorsal surface of the tongue and reaches receptors at a depth of 200–400 microns below the epithelium. Significant improvement before and after treatment has been demonstrated in the following measurement variables: Dynamic Gait Index (DGI), Activities-specific Balance Confidence Scale (ABC), Dizziness Handicap Inventory (DHI), and Sensory Organization Test (SOT).^45^

Level of Evidence: *B*.

Degree of recommendation: *recommended*.

### Surgeries for vestibular diseases

#### Labyrinthectomy and vestibular neurectomy:^46^

Procedures that may be used in selected cases of disabling vertigo refractory to clinical treatment. Labyrinthectomy is limited to patients with socially useless hearing, while vestibular neurectomy is one of the options for patients with useful hearing.

Level of Evidence: *C*.

Degree of recommendation: *optional*.

#### Endolymphatic sac decompression:^47^, ^48^

The purpose of the procedure is the symptomatic control of patients who are refractory to the clinical treatment of Meniere’s disease. However, no class or scientific society was found that adopted a recommendation for this subject. The procedure becomes an individual option of each professional, considering the characteristics of the case.

Level of Evidence: *C*.

Degree of recommendation: *optional*.

#### Occlusion of anterior semicircular canal dehiscence

It is a treatment option for the control of vertigo symptoms and is only indicated for patients with disabling symptoms of superior semicircular canal dehiscence (SSCD) syndrome. There is still no evidence to determine the most effective surgical technique. A systematic review published in 2017^49^ concluded that the surgical treatment of SSCD Syndrome has moderate effectiveness for disabling vertigo crises.

Level of Evidence: *C.*

Degree of recommendation: *optional*.

#### Fistula occlusion:^50^

It is a safe surgical treatment indicated for the management of patients with perilymphatic fistula, as long as its diagnosis is established.

Level of Evidence: C.

Degree of recommendation: *recommended*.

## Conclusion

The large amount of information presented in this article is a demonstration of the advancement of neurotology in recent years. The authors hope that this review may help the physician to understand, approach and treat patients with vestibular symptoms.

## Conflicts of interest

The authors declare no conflicts of interest
